# Emerging evidence of seed transmission of begomoviruses: implications in global circulation and disease outbreak

**DOI:** 10.3389/fpls.2024.1376284

**Published:** 2024-05-14

**Authors:** Nagamani Sandra, Bikash Mandal

**Affiliations:** ^1^ Seed Pathology Laboratory, Division of Seed Science and Technology, Indian Agricultural Research Institute, New Delhi, India; ^2^ Advanced Centre for Plant Virology, Division of Plant Pathology, Indian Agricultural Research Institute, New Delhi, India

**Keywords:** begomovirus, *Bemisia tabaci*, embryo, ELISA, PCR, seed, seed transmission, ToLCNDV

## Abstract

Begomoviruses (family *Geminiviridae*) are known for causing devastating diseases in fruit, fibre, pulse, and vegetable crops throughout the world. Begomoviruses are transmitted in the field exclusively through insect vector whitefly (*Bemisia tabaci*), and the frequent outbreaks of begomoviruses are attributed largely due to the abundance of whitefly in the agri-ecosystem. Begomoviruses being phloem-borne were known not be transmitted through seeds of the infected plants. The recent findings of seed transmission of begomoviruses brought out a new dimension of begomovirus perpetuation and dissemination. The first convincing evidence of seed transmission of begomoviruses was known in 2015 for sweet potato leaf curl virus followed by several begomoviruses, like bhendi yellow vein mosaic virus, bitter gourd yellow mosaic virus, dolichos yellow mosaic virus, mungbean yellow mosaic virus, mungbean yellow mosaic India virus, pepper yellow leaf curl Indonesia virus, tomato leaf curl New Delhi virus, tomato yellow leaf curl virus, tomato yellow leaf curl Sardinia virus, and okra yellow mosaic Mexico virus. These studies brought out two perspectives of seed-borne nature of begomoviruses: (i) the presence of begomovirus in the seed tissues derived from the infected plants but no expression of disease symptoms in the progeny seedlings and (ii) the seed infection successfully transmitted the virus to cause disease to the progeny seedlings. It seems that the seed transmission of begomovirus is a feature of a specific combination of host-genotype and virus strain, rather than a universal phenomenon. This review comprehensively describes the seed transmitted begomoviruses reported in the last 9 years and the possible mechanism of seed transmission. An emphasis is placed on the experimental results that proved the seed transmission of various begomoviruses, factors affecting seed transmission and impact of begomovirus seed transmission on virus circulation, outbreak of the disease, and management strategies.

## Introduction

The transmission of plant viruses through seeds is one of the critical means of virus perpetuation and dissemination that ensures the ecological existence of plant viruses (Baker, 1972; Albrechtsen, 2006; Hull, 2009). Seed transmission results through intricate interactions of the host and the virus (Bennet, 1969; Bos, 1977; Carroll, 1981). Bean common mosaic virus (BCMV) was the first plant virus showed to be seed transmitted on common bean (*Phaseolus vulgaris*) (Stewart and Reddick, 1917). Approximately more than one-third of known plant viruses have been reported to be seed transmitted in various fiber, food ornamental crops, and weeds (Singh and Mathur, 2004; Sastry, 2013). Some seed-transmitted RNA viruses such as barley stripe mosaic virus (BSMV), lettuce mosaic virus (LMV), pea seed-borne mosaic virus (PSbMV), potato virus Y, plum pox virus, tomato black ring virus (TBRV), raspberry ringspot virus (RRSV), and wheat streak mosaic virus were reported to cause huge crop losses from 10% to 98% (Stacie-Smith and Hamilton, 1988; Cambra et al., 2006; Valkonen, 2007). Seed transmission is an efficient survival strategy for plant viruses, especially for those with narrow host ranges and infects annual plant species. Several genera of RNA viruses, e.g., *Bromovirus*, *Carlavirus*, *Carmovirus*, *Comovirus*, *Cucumovirus*, *Fabavirus*, *Furovirus*, *Hordeivirus*, *Ilarvirus*, *Nepovirus*, plant *Rhabdovirus*, *Potexvirus*, *Potyvirus*, *Sobemovirus*, *Tobamovirus*, *Tobravirus*, and *Tymovirus* are known to be transmitted through seeds. The genera of DNA viruses such as *Caulimovirus* and *Badnavirus* are also known to be transmitted through seeds of the infected plants. However, seed-borne or seed transmission nature in the case of the DNA virus genus, *Begomovirus*, was not known. Recently, several begomovirus species have been reported to seed-borne in nature.

The genus *Begomovirus* (Family: *Geminiviridae*) is the largest genus of all plant virus families containing as many as 445 recognized virus species (Website: https://ictv.global/msl). The genome of begomovirus is single-stranded circular DNA (ssDNA), which is encapsidated inside quasi-icosahedral twin particles of 22 nm ×38 nm in size. Based on the number of the DNA components, begomoviruses are classified as monopartite containing a single DNA component and bipartite begomoviruses containing two DNA components, DNA-A and DNA-B (Fauquet et al., 2003; Lefkowitz et al., 2018). Both the DNA-A and DNA-B are approximately 2.8 kb in size. The DNA-A genome contains six open reading frames (ORFs), i.e., two are in virion sense (AV1 and AV2) and four are in complementary sense orientation (AC1, AC2, AC3, and AC4). The DNA-B consists of two ORFs, BV1 and BC1 in virion and complementary sense strand, respectively. In DNA-A, AV1/V1 codes for coat protein (CP), AV2/V2 for a protein of unclear function, AC1/C1 codes for a replication associated protein (Rep), AC2/C2 for a transcriptional activator protein (TrAP), and AC3/C3 encodes the protein replication enhancer (Ren), whereas AC4/C4 gene function is not yet known. In DNA-B, the BV1 encodes for a nuclear shuttle protein (NSP) responsible for nucleocytoplasmic transport of viral DNA and the BC1 for a movement protein (MP), required for intra- and intercellular movement of the viral DNA, respectively (Hanley-Bowdoin et al., 1999; Fiallo Olive et al., 2021). The noncoding region called intergenic region (IR, ~500 bp) contains the origin of replication (ori), where the viral Rep protein binds for commencing rolling circle DNA replication. A part of this region is common to both DNA-A and DNA-B of bipartite begomoviruses. The IR region also contains the promoter/regulatory elements for viral genes expression in both V-sense and C-sense strand (Arguello-Astorga et al., 1994). Monopartite begomoviruses are often associated with either alphasatellite or betasatellite DNAs of approximately 1.4 kb in size. The alphasatellites encode their own replication-associated protein (Rep), whereas betasatellite do not code for Rep protein but carry a single ORF (βC1), encoding multifunctional protein. Both alpha and betasatellites are dependent upon the helper virus for replication and, in several cases, attenuates the symptoms produced by helper virus (Idris et al., 2011).

The begomoviruses, in general, produce similar symptoms in plants like curling, leaf crumpling, leaf distortion and stunting, golden-light green yellow mosaic/mottle, mosaic, veinal, or interveinal yellowing and thereby causing severe yield losses. The diseases caused by begomoviruses are a serious threat to the production of many pulse crops, vegetables, root, and fiber crops in tropical, subtropical, and temperate regions of the world (Navas-Castillo et al., 2011). The economic impact of individual begomovirus epidemics can be enormous. For example, yield losses up to 96% have been reported for bhendi yellow vein mosaic virus (Pun and Doraiswamy, 1999). In leguminous crops especially blackgram, mungbean, and soybean, yield losses due to yellow mosaic disease (YMD) caused by begomoviruses have been estimated to be approximately $300 million per year (Varma and Malathi, 2003).

Seed-borne infection of begomoviruses is of new insight, and therefore, it has induced an excitement for the greater understanding of its validity and anticipated impact in epidemics of begomovirus diseases. This review focuses on begomovirus transmission process, evidence of various begomoviruses reported to be seed-borne or seed transmitted, external and internal factors affecting the seed transmission, and epidemiological significance of seed transmission on circulation and outbreak of begomovirus disease. Lastly, a brief account of management of seed-borne begomoviruses is also provided.

## *Begomovirus* transmission process

Begomoviruses are exclusively transmitted through whitefly, *Bemisia tabaci* (Genus: *Homoptera*, Family*: Aleyrodidae*), under natural conditions. In the experimental conditions, they are transmitted by the various methods, e.g., grafting, occasionally through infected leaf sap, biolistic delivery of direct viral DNA and agro-inoculation with the infectious clones (Stanley, 1983; Lazarowitz et al., 1989; Kikkert et al., 2004; Hassan et al., 2016). During late 1970s, begomovirus was known as geminivirus, a new class of plant infecting virus having unique twin-quasi-isometric virion morphology (Harrison et al., 1977a, b). Subsequently, three major groups of geminiviruses were discovered and then classified based on the differences in infection of monocot and dicot plant species and insect vector transmission: (i) Whitefly-transmitted geminiviruses infecting dicot plant species (WTGs), (ii) Whitefly-transmitted geminiviruses infecting monocot plant species, and (iii) leafhopper-transmitted geminiviruses infecting monocot plant species. During 1980s, WTGs were given a status of a genus, *Begomovirus* (created from the type species, bean golden mosaic virus) under the family *Geminiviridae* (van Regenmortel et al., 1997). *B. tabaci* complex corresponds to >40 morphologically indistinguishable species found in tropical and subtropical regions disseminates begomoviruses very efficiently, causing important damage to staple food crops, vegetables, and ornamentals worldwide (Czosnek, 2007; Lee et al., 2013; Mugerwa et al., 2018). In addition to *B. tabaci*, two other species of whiteflies have also been reported as vector of begomoviruses, *Trialeurodes ricini* transmitting tomato yellow leaf curl virus (ToYLCV) in Egypt (Idriss et al., 1997) and *T. vaporariorum* transmitting tomato leaf curl New Delhi virus (ToLCNDV) in India under glasshouse conditions (Sangeetha et al., 2018).

Begomovirus and whitefly have specific and interesting interactions that result in a circulative and persistent mode of vector transmission process. The translocation pathway of begomovirus in the whitefly body has been described well by Czosnek et al., 2017. Briefly, the process involves five major steps: ingestion of begomovirus by whitefly through stylet while feeding on the infected leaves, passing into hemolymph through midgut, entry in to primary salivary glands, secretion of begomovirus from secretory gland cells into central lumen, and then egestion back to plants. This circulatory process takes several hours to complete, and begomovirus persists in whitefly bodies for many days. This is how the whitefly transmission of begomovirus is referred as a circulative persistent mode of transmission. However, while persisting in the whitefly body, viral replication and transovarial transmissions of begomovirus is not clear yet, as the current studies have shown inconsistent results (Ghanim et al., 1998; Bosco et al., 2004; Becker et al., 2015; Sanchez-Campos et al., 2016; Fiallo-Olive and Navas-Castillo, 2019; Guo et al., 2018; Ghosh et al., 2021).

For many years, begomoviruses were known not be transmitted through the seeds originated from the virus-infected plants. Hence, it has been generally accepted that begomoviruses are not vertically transmitted through seed to the next generation of plants. However, recent reports showed that some of the begomoviruses were seed-borne, *viz*., bitter gourd yellow mosaic virus (BgYMV), dolichos yellow mosaic virus (DoYMV), mungbean yellow mosaic virus (MYMV), sweet potato leaf curl virus (SPLCV), tomato leaf curl New Delhi virus (ToLCNDV), and tomato yellow leaf curl virus (ToYLCV) (Kim et al., 2015; Kil et al., 2016; Kothandaraman et al., 2016; Kil et al., 2017, 2018; Sangeetha et al., 2018; Suruthi et al., 2018; Manivannan et al., 2019; Gomathi Devi et al., 2023). These studies have changed the previous understanding and brought the new perspective of interpretation of global circulation and outbreak of begomovirus diseases in agricultural crops.

## Past knowledge of *begomovirus* seed transmission

Begomoviruses are mostly confined to the phloem parenchyma, cambium, and rarely mesophyll parenchymatous tissues. In the absence of symplastic connection between mother plant cells and seed tissue, it used to be considered that the embryonic tissues were inaccessible to begomoviruses (Rojas et al., 2005; Kothandaraman et al., 2016). The seed transmission nature of abutilon mosaic virus (AMV) was initially expected since 1934 (Keur, 1934). Later, few preliminary studies were conducted regarding the begomovirus seed transmission before the first molecular evidence came into existence. Among those few studies are listed in **Table 1**.

**Table 1:** Past knowledge of begomovirus seed transmission.

S.No.	Virus	Crop	Country	Experiment/Remarks	Reference
**1.**	Abutilon mosaic virus (Variegation virus) (AMV)	Flowering maple (*Abutilon Thompsonii)*	West Indies	Examined the seeds of Abutilon species by grow out tests. Also observed the seedlings obtained by crossing *A. darwini* and *A. thompsonii* produced yellow flecks on leaves	Lindemuth (1907)
**2.**	Chrysanthemum indicum yellow vein Delhi virus (CiYVDV)	Chrysanthemum (*Chrysanthemum indicum*)	India	Progeny test along with PCR proved CiYVDV was neither seed-borne nor seed transmissible in nature	Marwal et al. (2013)
**3.**	Pepper yellow vein mali virus (PepYVMV)	Sweet Pepper (*Capsicum annuum*) and hot pepper (*C. frutescens*)	Barkina Faso, West Africa	Observed 1,000 seedlings raised from infected pepper fruit remain symptomless and concluded the absence of seed transmission without molecular evidence.	Fidele et al. (2008)
**4.**	Tomato yellow leaf curl virus (TYLCV)	Tomato (*Solanum lycopersicum*)	Israel	The differential distribution of TYLCV nucleic acids in roots, stems, leaves, flowers, and fruits was observed using squash blot procedure	Navot et al. (1989)
			France	Distribution of TYLCV was observed in stem, sepals, skin, pulp, and peduncle using TAS-ELISA. It was proved that TYLCV can be acquired and transmitted by *B. tabaci* from tomato fruit after 3 h AAP	Delatte et al. (2003)

## Emerging evidence for seed-borne nature of *begomoviruses*

Seed-borne and seed transmission terms often used synonymously. However, the mere presence of virus in the different parts of seed tissues does not always result in successful transmission from seed to seedlings. The first successful demonstration of seed transmission of begomovirus was reported in 2015 with sweet potato leaf curl virus (SPLCV) through sweet potato seeds (Kim et al., 2015). Subsequently, 10 more begomoviruses in different plant species were reported to be either present in the seed tissues but not transmitted to the progeny plant or to be successfully transmitted from seed to seedling causing disease symptoms (**Table 2**). The experimental results of seed-borne or seed transmission nature of these viruses are summarized below. Still there are a vast majority of begomoviruses yet to be assessed to understand if the seed transmission is a recently evolving phenomenon for begomoviruses. In addition to begomoviruses, seed transmission was also reported in the member of other genera of the family *Geminiviridae*, e.g., beet curly top virus and beet curly top Iran virus of the genus, *Becurtovirus* (Anabestani et al., 2017), and sweet potato symptomless virus 1 of the genus, *Mastrevirus* (Qiao et al., 2020).

**Table 2:** Recent studies documented seed transmission of begomoviruses.

S.No	Virus detected	Host species	Type of seed borne nature	Country	Reference
1.	Bhendi yellow vein mosaic virus (BYVMV)	Okra/Bhendi (*Abelmoschus esculentus*)	Seed-borne but not seed transmitted	India	Sisodia and Mahatma (2020)
2.	Bitter gourd yellow mosaic virus (BgYMV)	Bitter gourd *(Momordica charantia)*	Seed transmitted	India	Manivannan et al. (2019)
3.	Dolichos yellow mosaic virus (DoYMV)	Lablab bean (*Lablab purpureus*)	Seed transmitted	India	Suruthi et al. (2018)
4.	*Mungbean yellow mosaic virus* (MYMV)	Urd bean (*Vigna mungo*)	Seed transmitted	India	Kothandaraman et al. (2016)
			Seed transmitted	India	Niresh Kumar et al. (2023)
5.	Mungbean yellow mosaic India virus (MYMIV)	Yardlong bean (*Vigna unguiculata* subsp*. sesquipedalis*)	Seed transmitted	Indonesia	Mulyadi et al. (2021)
		Mungbean (*Vigna radiata*)	Seed-borne but not seed transmitted	India	Shobharani (2023)
6.	Pepper yellow leaf curl Indonesia virus (PepYLCIV)	Chili pepper (*Capsicum annuum*)	Seed transmitted	South Korea	Fadhilaa et al. (2020)
7.	Sweet potato leaf curl virus (SPLCV)	Sweet Potato (*Ipomoea batatas*)	Seed transmitted	South Korea	Kim et al. (2015)
			No seed transmission	USA	Andreason et al. (2021)
8.	Tomato leaf curl New Delhi virus (ToLCNDV)	Chayote (*Sechium edule*)	Seed transmitted	India	Sangeetha et al. (2018)
		Zuchini squash (*Cucurbita pepo*)	Seed transmitted	South Korea	Kil et al. (2020)
		Melon (*Cucumis melo* L.)	Seed-borne but not seed transmitted	Spain	Fortes et al. (2023)
		Bitter gourd (*Momordica charantia*)	Seed transmitted	India	Gomathi Devi et al. (2023)
		Cucurbits (*Citrullus lanatus, Cucumis melo*, *Cucurbita moschata*, *Cucurbita pepo* and *Cucumis sativus*)	Seed-borne but not seed transmitted	Spain	Christina et al. (2023)
9.	Tomato yellow leaf curl virus (ToYLCV)	Tomato (*Solanum lycopersicum*)	Seed transmitted	South Korea	Kil et al. (2016)
		Benth (*Nicotiana benthamiana*)	No seed transmission	China	Rosas-Diaz et al. (2017)
		White soybean (*Glycine max*)	Seed transmitted	South Korea	Kil et al. (2017)
		Sweet pepper (*Capsisum annuum*)			Kil et al. (2018)
		Tomato, *N. benthamiana*	Seed-borne but not seed transmitted	Spain	Perez-Padilla et al. (2020)
		Tomato variety Lanai	No seed transmission	USA	Rajabu et al. (2018)
10.	Tomato yellow leaf curl Sardinia virus (ToYLCSV)	Tomato (*Solanum lycopersicum*)	No seed transmission	Iran	Tabein et al. (2021)
11.	Tomato mottle virus (ToMoV)	Tomato (*Solanum lycopersicum*)	No seed transmission	USA	Rajabu et al. (2018)
12.	Tomato golden mosaic virus (TGMV)				
13.	Okra yellow mosaic Mexico virus (OYMMV)	Roselle (*Hibiscus sabdariffa* L.) and weeds (*Sida* species)	Seed transmitted only in weed species	Mexico	Ortega et al. (2018)

a. Bhendi yellow vein mosaic virus (BYVMV), a monopartite begomovirus, was first reported from India in bhendi (*Abelmoschus esculentus*) crop showing the symptoms of vein clearing and yellowing with reduced size of leaves and fruits (Kulkarni, 1924). BYVMY was reported to be responsible for 96% yield loss (Pun and Doraiswamy, 1999). The seed transmission nature of BYVMV was studied in bhendi cultivar Gujarat Okra-2 by establishing virus infection through side veneer grafting method with IN-NVS-2018 virus isolate. BYVMV was detected through conventional polymerase chain reaction (PCR) using specific primers in both vegetative and reproductive tissues, *viz*., leaf, flower bud, petal, sepal, ovary, pollen, fruit, seed coat, cotyledon, and embryonic axes. In grow-out test, none of the seedlings showed virus-specific symptoms and tested negative for the BYVMV through PCR, indicating the non-seed transmission nature (Sisodia and Mahatma, 2020). The result suggests that BYVMV was present in part of the seed or reproductive tissues, but the virus could not be successfully transmitted from infected seed to seedling.

b. Bitter gourd yellow mosaic virus (BgYMV) is a bipartite begomovirus that causes yellow mosaic disease (YMD) on bitter gourd (*Momordica charantia*) with the symptoms of yellowing, puckering, and stunting (Raj et al., 2005a). Recently, the coccinia mosaic Virudhunagar virus was also renamed as BgYMV while studying its seed transmission nature in bitter gourd (Manivannan et al., 2019; GenBank Acc no. KY860899). The seed transmission of BgYMV was confirmed through double antibody sandwich enzyme-linked immunosorbent assay (DAS-ELISA) and PCR by detecting from different fruit and seed parts, *viz*., fruit rind, fruit pulp, whole seed, seed coat, endosperm, embryo, and also from progeny seedlings raised from infected seeds. Higher absorbance values were observed in fruit rind, whole seed, and seed coat (OD_405nm_, 0.71–1.89) compared to fruit pulp, endosperm, and embryo (0.30–0.64). The seed infectivity was found to be 79.16%, and transmission rate from seed to seedling was approximately 32.05%. The interesting feature of BgYMV seed transmission study was the observation of mild mosaic and yellow mottling symptoms in the raised seedlings from infected seed, which intensified to puckering and yellow discoloration in further grow-out test (Manivannan et al., 2019).

c. Dolichos yellow mosaic virus (DoYMV) is a bipartite begomovirus responsible for YMD in legume species with the symptoms of faint chlorotic specks and bright yellow mosaic patches (Bisaro, 1996; Akram et al., 2015). The full genome characterization of DoYMV was performed from Kanpur, India (KJ481204; Akram et al., 2015). The seed transmission nature of DoYMV was studies with the TN-TM1 isolate from Tamil Nadu, India in lablab bean (*Lablab purpureus*), where the virus was experimentally detected through DAS-ELISA and PCR analysis of whole seed, seed parts, and progeny seedlings (Suruthi et al., 2018). In the seed collected from infected plants, the virus was detected up to 100% in embryonic axis followed by 37.5% and 69.23% in seed coat and endosperm, respectively. Even though, the seedlings did not display any symptoms in grow-out test, the virus could be detected up to 46% and 55% through DAS-ELISA and PCR, respectively. DoYMV is an interesting example, where virus is present in tissues of seed originating from the infected plants and in the seedlings raised from such infected seeds. With the available experimental data, the lack of symptom expression in the virus containing seedlings of lablab bean is hard to explain. However, if this biology is consistent, DoYMV-TN-TM1 is a unique example of latent seed transmission, where the disease inciting ability was retarded to latency while passaging from seed to seedling.

d. Mungbean yellow mosaic virus (MYMV) is a bipartite begomovirus first reported from mung bean plants showing yellow mosaic symptoms during 1950s in India (Nariani, 1960). The typical symptoms of the disease are bright yellow mosaic of leaves, stunted growth, reduction in leaf lamina and pod number, along with highly misshapen shrivelled seeds. Small and deformed pods with presence of yellow spots on harvested seed from susceptible black gram (*Vigna mungo*) genotypes gave the preliminary clue for the presence of MYMV in the seeds (Nariani, 1960; Williams et al., 1968). Mahatma and Pawar (2015) tested infected mung bean (*Vigna radiata*) plants [Cultivar (cv) GM-4] for the presence of MYMV and its distribution in various reproductive parts, namely, whole flower, floral parts, empty pod, whole seed, and seed parts through PCR. MYMV detection was in the range of 40%–100% in all the observed parts except gynoecium and embryonic axis. The virus was also detected in the callus developed from the MYMV-infected cotyledon. However, virus was not transmitted from seed to seedlings in grow-out test and concluded that MYMV is seed-borne but not seed-transmitted in nature. On the contrary, the seed transmission nature of MYMV was proved in a field infected with black gram cv. Co-5 from Tamil Nadu, India (Kothandaraman et al., 2016). The presence of MYMV in whole seeds, seed coats, cotyledon, and embryonic axis was confirmed through confocal microscopy, DAS- ELISA, immunosorbent electron microscopy, PCR, sequencing, and Southern and dot blot hybridization tests. The seed to seedling transmission rate was observed to be 32% through DAS-ELISA and PCR, although the seedlings remained symptomless in grow-out test. However, whitefly transmission of MYMV was not demonstrated from the PCR-confirmed symptomless seedlings. Niresh Kumar et al. (2023) studied the seed-borne nature of MYMV in blackgram susceptible (Co-5) and resistant cultivars (Mash 114) through whitefly-mediated transmission. The whiteflies after feeding the MYMV-susceptible cultivar released on to the cvs Co-5 and Mash 114. After 20 days of post-inoculation (dpi), the Co-5 plants showed the symptom expression, whereas Mash 114 did not display any symptoms. PCR amplification was observed in different seed parts like seed coat, cotyledon, and embryonic axis of Co-5, whereas amplification was observed only for seed coat and cotyledon but not with embryonic axis of resistant cultivar Mash 114 with the DNA-A and DNA-B specific primers. This study confirmed the seed transmission nature of MYMV in susceptible cultivar.

e. Mungbean yellow mosaic India virus (MYMIV) is a bipartite begomovirus also responsible for YMD in leguminous crops and confined to northern, central, and eastern regions of the India, Pakisthan, Bangladesh, Nepal, and Vietnam (Malathi and John, 2009). Naimuddin et al. (2016) studied the seed-borne nature of MYMIV in mungbean genotype T44 plants displaying yellow patches on the harvested seed. The PCR analysis of symptomatic mungbean seed using MYMIV specific primers revealed the presence of virus in the whole seed. However, there was no proper experimental proof regarding the seed to seedling transmission of MYMIV. Later, seed transmission nature of MYMIV was reported from yardlong beans (*Vigna unguiculata* subsp. *sesquipedalis* L.) using certified commercial seeds and malformed pods collected from infected plants in Indonesia (Mulyadi et al., 2021). PCR analysis using MYMIV specific and universal begomovirus primers showed the amplification from whole seed, seed coat, cotyledons, and young leaves from sprouting seeds. The seedlings in the progeny test showed yellow mosaic symptoms at sprouting stage and vein clearing and yellow mosaic symptoms at 14 and 21 days after sowing (DAS), whereas leaf blades turned bright yellow by 45 DAS, confirming MYMIV seed transmission nature. In another study, MYMIV was found to be associated with whole seed and seed coat but not with cotyledons and embryonic axes through ELISA and PCR in mungbean cvs Pusa 9531 and Pusa 1371. No visual symptoms were observed in seedlings raised from infected seed for three consecutive seasons, viz., Spring–Summer, Kharif 2021, and Spring–Summer 2022 (Shobharani, 2023).

f. Pepper yellow leaf curl Indonesia virus (PepYLCIV) is a bipartite begomovirus responsible for pepper yellow leaf curl disease (PepYLCD) observed in many pepper (*Capsicum annuum* L.)-growing regions of Indonesia (Sulandari et al., 2007). PepYLCIV generally produce the symptoms of leaf curling, faint chlorotic specks on leaf lamina, which later develop into bright yellow mosaic patches and distorted leaves (Rusli et al., 1999). The first complete genome sequence of PepYLCIV was determined from pepper, tomato, and ageratum (Tsai et al., 2006; Sakata et al., 2008). PepYLCD was initially thought to be transmitted by grafting and whiteflies but not through seed or by mechanical inoculation (Rusli et al., 1999; Sulandari et al., 2007). The seed transmission nature of PepYLCIV was established in chili pepper plants showing symptoms of typical leaf curling, yellowing, blisters, and dwarfism in Indonesia through Uracil DNA glycosylase-PCR (UDG-PCR). The PepYLCIV was detected through UDG-PCR in symptomatic leaf samples, whole seed, cotyledons, hypocotyls, radical, and embryo. Seedlings raised from the infected chili pepper seed in grow-out test showed the presence of 25%–67% of PepYLCIV DNA-A and 50%–100% of DNA-B through UDG-PCR. The occurrence of replication and systemic viral movement was confirmed through detection of DNA-A and DNA-B in specific parts of seedlings, *viz*., radicle, hypocotyl, and cotyledon (Fadhilaa et al., 2020). This study clearly showed the seed transmission nature of PepYLCIV through detection of DNA-A and DNA-B in seeds, embryos, and separate seedling organs derived from infected chilli pepper plants.

g. Sweet potato leaf curl virus (SPLCV) is a monopartite begomovirus responsible for leaf curl disease in sweet potato [*Ipomoea batatas* (L.) Lam] with the symptoms of upward leaf curling along with swelling veins, first reported from Taiwan and Japan (Cohen and Loebenstein, 1991). The SPLCV transmission generally occurs through propagation slips generated from infected sweet potato and whitefly *B. tabaci* (Valverde et al., 2004; Clark and Hoy, 2006; Simmons et al., 2009). The seed transmission nature of SPLCV in sweet potato cultivars Mokpo-69 and Singeon-mi was established through PCR, sequencing, and Southern blot hybridization from South Korea (Kim et al., 2015). Initially, the presence of SPLCV was confirmed from petals, floral tissues, seeds, and seedlings using IR specific primers. More than 70% of the harvested seed from SPLCV-infected sweet potato plants tested positive for SPLCV through PCR. SPLCV was also detected in whole dry seeds, seed coat, endosperm, and embryo using CP specific primers. The seed to seedling transmission rate was reported to be 21.21% and further confirmed the SPLCV replication in seedlings through Southern blot hybridization. On the contrary, in another study, Andreason et al. (2021) concluded that SPLCV was not seed transmissible. They observed the presence of SPLCV only on the seed coat but not on any new germinated cotyledons after testing the infected seeds in Petri dishes. The evaluation of the maternal genotype (USDA-10-102) for the SPLCV distribution through real-time and end-point PCR revealed the presence of SPLCV in all maternal tissue types including storage root, flower, and seeds. Large-scale evaluation of sweet potato seedlings from SPLCV contaminated seeds over 4 consecutive years (2016–2021), i.e., approximately 23,034 seedlings of 118 genotypes entries in insect proof greenhouse or growth chamber, showed negative result for seed transmission when tested through quantitative PCR (qPCR). Based on the large-scale grow-out test, seed coat and cotyledon test, and vector transmission experiments, it was concluded that SPLCV was not seed transmitted in sweet potato (Andreason et al., 2021).

h. Tomato leaf curl New Delhi virus (ToLCNDV) is a bipartite begomovirus responsible for total yield loss in tomato cultivation (Moriones et al., 2017; Zaidi et al., 2017). After the first report of tomato leaf curl disease from India, 48 begomovirus species have been reported to cause tomato leaf curl disease (Vasudeva and Sam, 1948; Padidam et al., 1995). The studies on seed transmission nature of ToLCNDV conducted in various crops, *viz*., bitter gourd, chayote, melon, zucchini squash, and other cucurbits are summarized below.

- Chayote (*Sechium edule* Sw.): chayote is an important cucurbitaceous vegetable crop grown widely in the hilly regions of India. The seed transmission studies of ToLCNDV in chayote were conducted through PCR, sequencing, and grow-out tests (Sangeetha et al., 2018). ToLCNDV produces the symptoms of yellow mosaic, curling, enation, and leaf distortion in chayote. ToLCNDV was detected in fruits and seed tissues (pericarp, mesocarp, seed coat, endosperm, and embryo) using CP specific primers through PCR. The sequencing of the rolling circle amplification (RCA) product from embryonic tissue DNA shared 95% identity with sequence of ToLCNDV obtained from the leaf sample. The seedlings raised from the infected chayote seeds were free of yellow mosaic symptom initially and developed dark green and leathery foliage after 60 DAS. Testing of progeny seedlings through PCR showed positive amplification of ToLCNDV, indicating the presence of viral genome in these seedlings.
- Zuchini squash (*Cucurbita pepo*): ToLCNDV causes symptoms of severe curling, yellow mosaic, and vein thickening of young leaves, stunted growth, rough skin, and reduced size fruit in Zuchini squash (Panno et al., 2016). The seed transmission nature of ToLCNDV (i.e., ToLCNDV ES) was studied in Zucchini squash cvs Ortano and Milos (Kil et al., 2020). Initially, the presence of ToLCNDV was confirmed from the whole seeds and naturally germinated seedlings from infected zucchini fruits under field conditions using AC1 specific primers. Later, the infected seeds were allowed to germinate under laboratory conditions. PCR analysis of DNA extracted from seed coat and germinated seedlings showed detection of ToLCNDV up to 61.36% of the test sample. Mechanical inoculation of homogenized sap on healthy plants prepared with raised seedlings from the infected seeds resulted in the infection with ToLCNDV, which was confirmed through PCR. All these results strongly confirmed the vertical transmission of ToLCNDV in Zucchini squash. Interestingly, the ToLCNDV ES found to be seed transmissible belonged to the subgroup I of ToLCNDV ES isolates (Parrella et al., 2018; Troiano and Parrella, 2023).
- Melon (*Cucumis melo* L.): Fortes et al. (2023) studied the seed-borne nature of ToLCNDV isolate ‘Spain’ strain in melon cvs Brimos, Mayor, and Nesta through agro-inoculation. The detection of ToLCNDV in floral parts and mature seed (seed cotyledons and embryo) through PCR with DNA-A specific primers revealed the presence of virus in seed. qPCR analysis of seed cotyledons and embryo revealed the low-level presence of ToLCNDV, indicating the viral contamination or infection of internal portions of seed. Chemical disinfectant treatment of melon seeds significantly reduced the detectable virus, suggesting contamination of the external portion of melon seed coat with ToLCNDV. Analysis of progeny seedlings germinated from ToLCNDV-infected melon seeds through hybridization, conventional PCR, and qPCR did not show any evidence to support the seed transmission nature of ToLCNDV.
- Bitter gourd (*Momordica charantia*): The seed transmission nature of ToLCNDV in market- and field-collected samples of bitter gourd hybrids H1, H2, H3, H4, and Co-1 were studied through DAS-ELISA, PCR, and grow-out test (Gomathi Devi et al., 2023). The virus was detected up to 63%, 26%, 20%, and 10% in hybrids H1, H2, H3, and H4, respectively, for market-procured seeds. PCR analysis of seeds with ToLCNDV specific primers showed the infection level of 76% in market-collected hybrids compared to field samples. Analysis of progeny seedlings through PCR showed 3%–5% seed transmission for ToLCNDV with the H1, H2, and H3 market seed. Whitefly transmission studies from symptomatic and asymptomatic plants in a micro-plot experiment revealed that seed-borne virus can act as potential inocula as there was 43.3% initial seed transmission, whereas it increased to 70% after release of 60 whiteflies.
- Cucurbits: Christina et al. (2023) evaluated the seed transmission nature of ToLCNDV mediterranean isolate in cucurbits like cucumber (*Cucumis sativus*), musk melon (*Cucumis melo*), winter squash (*Cucurbita moschata*), zucchini (*Cucurbita pepo*), and watermelon (*Citrullus lanatus*) through conventional PCR, qPCR, progeny assay, Southern blot hybridization, and RCA. ToLCNDV was detected in leaf tissues, petals, stamens, pistils, and seed parts of *C. melo* genotypes with peculiar symptoms. Whereas *C. moschata* genotypes did not display any symptoms, ToLCNDV was detected in all the tested parts except endosperms. There was a significant reduction in the accumulation of ToLCNDV in bleach-treated endosperm and embryo tissues compared to untreated seeds or the treated seed coats. An interesting feature of this study was that *C. melo* and *C. moschata* offsprings did not display any symptoms, but virus-specific amplification was observed at 30 days and 60 days of post-germination. However, replicative forms of ToLCNDV were not observed from progeny seedlings through southern hybridization and RCA. ToLCNDV was also not detected in the 30 commercial watermelon seed lots evaluated, whereas it was observed in one sample each out of 43 and 19 genotypes of cucumber and zucchini squash, respectively. Based on these results, it was concluded that ToLCNDV is seed-borne but not seed transmitted in nature.

i. Tomato mottle virus (ToMoV) and tomato golden mosaic virus (TGMV) are the bipartite begomoviruses responsible for severe disease in tomato with the symptoms of brilliant chlorotic yellowish mottle of the foliage and golden mosaic (Stein et al., 1983; Agrios, 2005). The seed transmission of ToMoV and TGMV was studied in tomato cultivar ‘Florida Lanai’ through agro-inoculation. The progeny seedlings raised with infected seed collected from agro-inoculated plant fruits neither display any peculiar symptoms nor showed amplification with virus specific primers in PCR (Rajabu et al., 2018). These results confirmed the absence of seed transmission for ToMoV and TGMV.

j. Tomato yellow leaf curl virus (TYLCV) is a widely spread monopartite begomovirus, responsible for tomato yellow leaf curl disease (TYLCD) and severely limiting tomato (*Solanum lycopersicum* L.) cultivation in warm and temperate regions of the world (Moriones and Navas-Castillo, 2000; Rybicki, 2015). The symptoms of the TYLCD include leaf curling and yellowing, stunting of plant and flower abortion (Hanssen et al., 2010). Infected plants produce fruit with reduced market value, whereas infection during early growth stages can lead to total yield loss (Moriones et al., 2011). Since the first report of the virus from Middle East in 1931, TYLCV has spread continuously throughout tropical and subtropical areas (Czosnek and Laterrot, 1997). Kil et al. (2016) produced the first molecular evidence for seed transmission nature of TYLCV with Israel Isolate (TYLCV-IL) in tomato. Initially, the presence of TYLCV was confirmed in newly germinated tomato seedlings from seeds of infected tomato fruit through PCR under field conditions. Later, agro-inoculation with TYLCV-IL infectious clone and whitefly (*B. tabaci*, Q biotype)-mediated inoculation with TYLCV-IL was performed on susceptible (Seogwang) and resistant (Bacchus) tomato cultivars. TYLCV was observed in vegetative, floral, and seed tissues of infected tomato plants and in the seedlings germinated from the infected seeds through PCR with the detection level of 20%–100%. The average seed to seedling transmission rate was reported to be 84.62% and 80.77% with whitefly and agro-mediated transmission, respectively. Whitefly transmission of TYLCV was also observed from symptomatic and asymptomatic seedlings raised from infected seeds to healthy tomato plants, which strengthen the concept that offsprings from infected tomato plant can act as a source of inoculum for *B. tabaci*-mediated transmission. Just et al. (2017) demonstrated the localization of TYLCV DNA and transcripts of the CP gene specifically to the phloem tissue of young fruit, sepals, petals, and embryo of developing tomato seed through *in situ* hybridization experiments. The accumulation of TYLCV with increasing titers was observed in early developing tomato fruit tissues from anthesis until 21 days post-anthesis through qPCR (Kil et al., 2017, 2018). reported TYLCV-IL seed transmission in asymptomatic hosts, *viz.*, sweet pepper (*Capsicum annum* L.) and white soybean (*Glycine max* L.) plants, through agro-inoculation, which were previously considered to be non-host species for TYLCV-IL. The results from this study for the first time indicated that asymptomatic hosts could act as a reservoir of TYLCV, which could infect tomatoes. However, there was no mention regarding seed to seedling transmission percentage. Rosas-Diaz et al. (2017) studied the seed-borne nature of TYLCV in the experimental host *Nicotiana benthamiana* through agro-inoculation. Inoculated plants displayed the symptoms of stunting and leaf curling. The seedlings raised from TYLCV-infected *N. benthamiana* seeds were agro-inoculated continuously for second and third generation. The qPCR analysis of leaf and seed tissues from third generation with TYLCV specific primers showed the accumulation only in leaf but not in seed tissues. These results revealed that TYLCV was not seed transmitted in *N. benthamiana* despite the higher virus accumulation observed in leaf tissues. Perez-Padilla et al. (2020) evaluated the seed transmission nature of two TYLCV-IL isolates in susceptible seven tomato genotypes (six Spanish land races and one cultivar) and *N. benthamiana.* The seed transmission nature of TYLCV was studied through hybridization of tissue blots, PCR, qPCR, monitoring of fluorescence, and grow-out test. Initially, the presence of TYLCV in field infected tomato plants and agro-inoculated tomato and *N. benthamiana* plants along with the seed was confirmed through PCR and qPCR using TYLCV specific primers. Monitoring of TYLCV in infected tomato and *N. benthamiana* plants using fluorescent proteins revealed the presence of TYLCV in petals, stamens, style, and ovary tissue of tomato, whereas fluorescence was observed only in the ovary septum and silique of *N. benthamiana*, indicating the close association of TYLCV with the seed during maturation. The qPCR analysis of tomato seed revealing the presence of high viral DNA load in non-disinfected tomato seed compared to surface disinfected tomato seed suggests that most of the virus is located externally as contaminant of the seed coat. Progeny seedlings grown either from field infected or artificially infected tomato and *N. benthamiana* plants did not display any TYLCV characteristic symptoms and testing through hybridization and qPCR, indicating no seed to seedling transmission. Transmission test through whiteflies from progeny seedlings to healthy seedlings did not result in any symptoms even after 60 days of inoculation access period. Transmission assays carried out with seven tomato genotypes and more than 3,000 tomato plants revealed no evidence of seed transmission from surface disinfected or untreated seed for two TYLCV-IL Mediterranean isolates in tomato and *N. benthamiana.* These results support the evidence that TYLCV-IL is seed-borne but not seed transmitted in tomato and *N. benthamiana.*

k. Tomato yellow leaf curl Sardinia virus (TYLCSV) is an important monopartite begomovirus responsible for worldwide spread of TYLCD in association with TYLCV and other TYLCV-like viruses (Moriones and Navas-Castillo, 2000). The seed transmission nature of TYLCSV was studied in highly susceptible tomato cultivar Money Maker through agro-inoculation with 1.8mer TYLCSV construct (Tabein et al., 2021). The agro-inoculated plants showed the typical symptoms of TYLCSV, *viz*., severe leaf curling, cupping, and yellowing on newly emerged leaves. The PCR analysis of DNA extracted from leaf, sepals, petals, pistils, stamens, fruit flesh, seeds, and embryos with TYLCSV specific primers (TY1/TY2) resulted in positive amplification, indicating the presence of virus in vegetative and reproductive tissues. The quantification of viral DNA through qPCR revealed that there was no statistically significant difference among different organs except whole seeds and embryos, which consisted of 10–10^3^ times less viral DNA compared to leaf tissues, respectively. Furthermore, Southern blot hybridization revealed the presence of genomic and replicative forms (ssDNA and dsDNA, respectively) in all extracts except seed and embryos. Interestingly, no RCA and amplification in PCR were observed with the DNA extracted from embryos after surface sterilization. Progeny seedlings raised with the seed collected from agro-inoculated plants did not display any symptoms of TYLCSV, and DNA was not able to be detected through Southern blot hybridization and no amplification was observed by PCR. These results clearly indicated that TYLCSV is not seed transmitted in tomato and detection in embryos was mainly due to surface contamination by TYLCSV DNA originating from the surrounding maternal tissue during seed formation.

Therefore, from the above experiments, it is clearly evident that transmission through seed might not be a general property for TYLCV and TYLCSV.

l. Okra yellow mosaic Mexico virus (OYMMV) is monopartite begomovirus responsible for vein clearing, mosaic, and yellowing symptoms in okra (*Abelmoschus esculentus*) and roselle (*Hibiscus sabdariffa* L.) (Velazquez-Fernandez et al., 2016). The seed transmission of OYMMV and whitefly-associated begomovirus 3 (WfaBV 3) was studied in roselle and roselle-associated weeds, *viz.*, *Sida acuta*, *S. Aggregate*, *S. collina*, *S. haenkeana*, and *Malacra fasciata* (Ortega et al., 2018). In this study, WfaBV3 and OYMMV were not detected by PCR in the different roselle cultivars from both seeds and seedlings. Furthermore, no RCA was observed from the infected seed, which supports the non-transmission of WfaBV3 and OYMMV by roselle seed. However, OYMMV was detected through PCR in seeds and progeny seedlings of all the weed species, although they did not display any symptoms. These results suggested that OYMMV was not seed transmitted in roselle but seed transmitted in weed species.

## Mechanism of seed transmission: *begomovirus* verses other plant viruses

**Figure 1:**
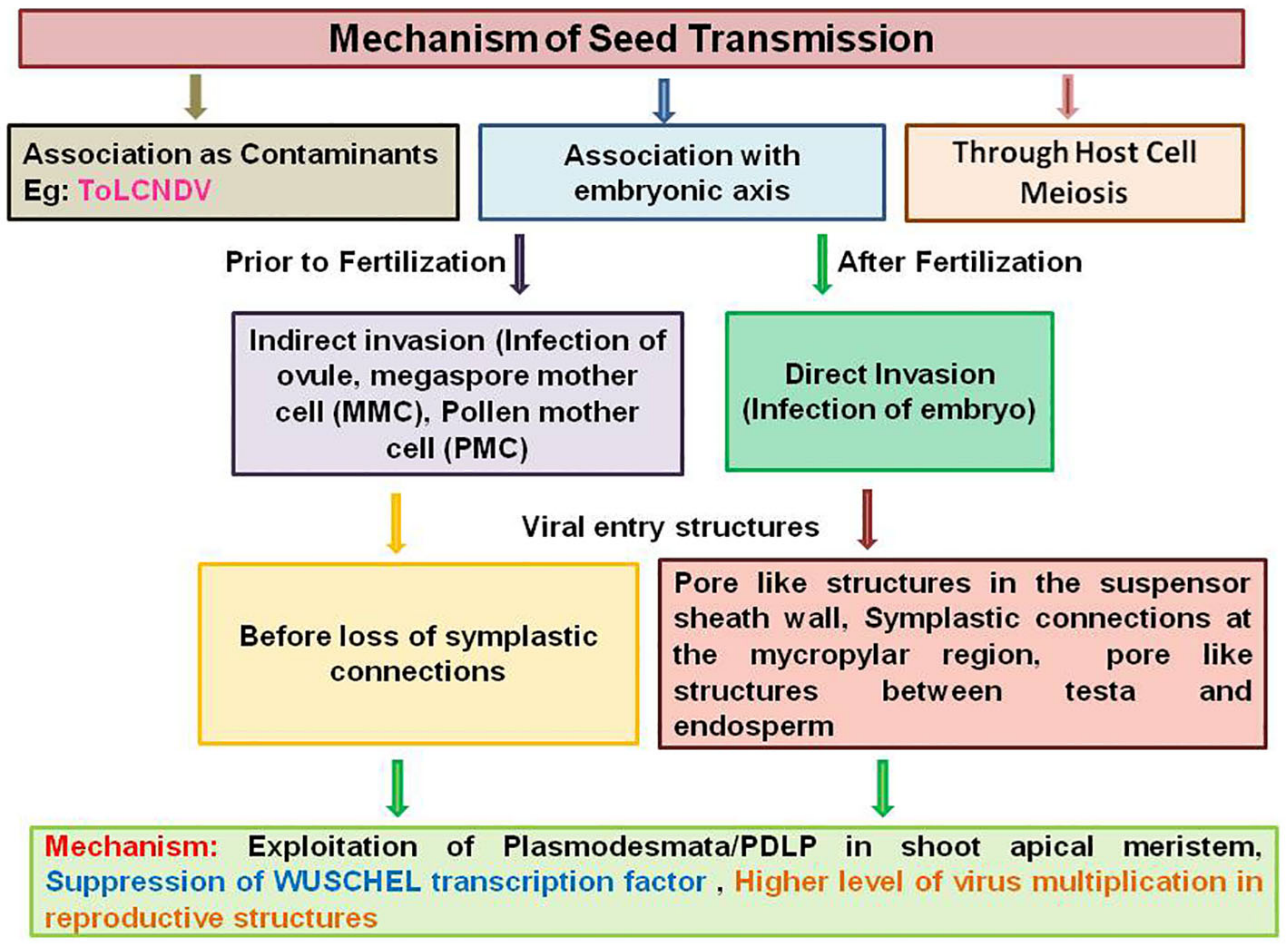
Schematic representation of seed transmission mechanism in plant viruses by associating as contaminants with the embryonic axis and through host cell meiosis.

Seed transmission of plant viruses occurs mainly by associating as contaminants with the embryonic axis and through host cell meiosis (**Figure 1**). Seedling infection occurs during germination when the plant virus present as contaminant on the seed coat or in the seed parts as reported in tobamoviruses and southern bean mosaic virus (SBMV) (*Sobemovirus*) (Hull, 2014; Dombrovsky and Smith, 2017). The case may be similar with mechanically transmissible begomovirus ToLCNDV, as sodium hypochlorite disinfectant treatment significantly reduced the detectable virus associated with melon seeds (Fortes et al., 2023). The most common true to type virus seed transmission occurs when virus infects an embryo during seed formation either directly or indirectly (Bennet, 1969; Timmerman et al., 2009). In an indirect invasion mechanism, infection of ovule, megaspore mother cell (MMC), and pollen mother cell (PMC) occurs prior to fertilization by altering the size exclusion limit (SEL) of plasmodesmata, whereas in direct invasion mechanism, infection of embryo occurs after fertilization through the suspensor (Maule and Wang, 1996; Oparka et al., 1997; Hull, 2014). Thus, most of the seed-transmitted viruses are carried within the embryo (Albrechtsen, 2006). The indirect mechanism of virus entry was reported for BSMV, where the virus enters early-developing barley female gametophyte (embryosac) and male gametophyte (pollen grain) before loss of symplastic connections to ensure seed transmission (Carroll and Mayhew, 1976a, b). Seed transmission through pollen is also a successful dissemination strategy reported for many viruses despite of the negative influence on pollen performance in terms of pollen production, pollen germination, and pollen tube growth (Bennet, 1969; Yang and Hamiiton, 1974; Amari et al., 2007; Matsushita et al., 2018). Direct infection of embryo through the suspensor was reported for PSbMV on pea cultivar (Wang and Maule, 1992, 1994; Roberts et al., 2003). Detailed studies regarding PSbMV seed transmission revealed the presence of pore-like structures in the suspensor sheath wall at the boundary between the endosperm and suspensor and symplastic connections at the micropylar region and at the boundary between the testa and endosperm, which facilitates the virus movement to the embryo (Roberts et al., 1998, 2003; Dombrovsky and Smith, 2017). It is obvious to investigate whether these types of routes can promote the begomovirus entry into the embryo or not. The third mechanism of seed transmission through host cell meiosis has been proposed for persistent viruses like *Oryza sativa endornavirus*, which achieves 100% seed transmission even if they do not move systemically (Boccardo et al., 1987; Moriyama et al., 1996; Fukuhara, 2019). However, the mechanisms by which these routes of entry are determined remain unclear.

Although, the potential significance of seed transmission of plant viruses was first recognized approximately 103 years ago, the specific mechanism by which some plant viruses are seed transmitted through seed while others are excluded is not yet known (Doolittle and Gilbert, 1919; Reddick and Stewart, 1919). The successful seed transmission depends on the factors like the ability of the virus to reach and invade gametic tissues, i.e., speed of virus movement and multiplication, gamete and embryo survival in the presence of the virus, seed production upon virus infection, and virus survival during seed maturation and storage (Lipsitch et al., 1995, 1996; Maule and Wang, 1996). For efficient seed transmission, virus should enter the vascular tissue to establish systemic infection followed by long distance movement through a series of cells like mesophyll, perivascular and phloem parenchyma, companion cells, and finally into the sieve tube elements (Astier et al., 2007). From systemic infection, viruses must make an entry into flower primordia to infect reproductive structures, which can be achieved by invading the shoot apical meristem (SAM) (Bennet, 1969; Maule and Wang, 1996). The presence of plasmodesmata between meristematic cells and expression of PDLP (Plasmodesmata Located Protein) family was observed in the SAM of *Arabidopsis* (Bayer et al., 2008; Kitagawa and Jackson, 2017). These connections might be potentially exploited by viruses to reach the reproductive structures during development. A recent study in *Arabidopsis* provided evidence that removal of WUSCHEL (WUS), a meristem-defining transcription factor, causes several RNA viruses to invade the meristem (Wu et al., 2020). Thus, the viral proteins may target WUS to suppress and achieve meristem invasion. It is also assumed that viral protein accumulation will be reduced to a level below the threshold required for symptom induction so that virus can escape host surveillance mechanism during meristem invasion (Paudel and Sanfaco, 2018). Few viruses can suppress the host defense mechanism RNAi as a viral strategy for meristem invasion mostly through specific viral silencing suppressor (VSR) proteins as observed for tobacco rattle virus (16K VSR) and CMV (2b protein) (Hernandez and Baulcombe, 2008; Sunpapao et al., 2009). The similar type of studies has to be carried for begomoviruses, which may shed some light on the mechanism of begomovirus seed transmission.

Wang and Maule (1994) suggested that a higher level virus multiplication in the reproductive structures favor gametophyte or embryo invasion by promoting virus crossing of the boundary between the maternal and progeny tissues. After invasion of reproductive tissues, virus should replicate and survive without damaging the physiological modifications during seed maturation (Gasparro et al., 2016). It means that a higher level of virus multiplication in the inflorescence may result in the higher percentage of seed transmission as observed for TYLCV-IL in tomato and CMV in *Arabidopsis* (Kil et al., 2016; Cobos et al., 2019). However, the virus inactivation may also happen during seed maturation as observed for alfalfa mosaic virus (AlMV), soybean mosaic virus (SMV), and SBMV (Uyemoto and Grogan, 1977; Bowers and Goodman, 1979; Bailiss and Offei, 1990). Thus, during virus seed transmission, viruses are physically and chemically attacked by host defense mechanisms such as callose deposition at plasmodesmata, RNA silencing, and natural immunity.

The ability of the virus to invade, multiply, and survive in reproductive tissues might be under the control of plant and virus genetic determinants through specific interactions. The host genetic determinants are poorly understood in the process of virus seed transmission and still not yet known in begomoviruses. There is very little information available on the role of plant genes involved in seed transmission of RNA viruses. In soybean, seed transmission of SMV was shown to be controlled by host genes homologs of *Arabidopsis* DCL3 (Dicer-like 3) and RDR6 (RNA-dependent RNA polymerase), which are components of RNA silencing plant defense response (Domier et al., 2011). In *Arabidopsis* infected with CMV, genome-wide association studies (GWAS) have identified genes *CIPK2* (Protein Kinase associated to calcineurin B-like protein) and *MAC5C* (MOS4-Associated complex subunit 5C protein) as determinants of CMV virulence and seed transmission rate (Montes et al., 2021). These two proteins were found to induce local resistance to the virus in flowers, thereby preventing the seed invasion and minimizing the effects of infection in seed production.

The different isolates of a particular seed-borne virus may have different rates of seed transmission in the same host, indicating the presence of viral determinants of seed transmission (Bennet, 1969; Shepherd, 1972). Virus genetic determinants involved in seed transmission were observed to be associated with regular functions like virus multiplication, movement, and invasion of plant reproductive organs (Cobos et al., 2019). Pseudorecombination studies with isolated viral RNAs have been used to link viral seed transmission phenotype to RNA1 of TBRV, RRSV, and CMV (Hanada and Harrison, 1977; Hampton and Francki, 1992). In BSMV, primary genetic determinants of seed transmission were mapped to the 5′ untranslated leader of RNAγ, a 369-bp repeat in the γa gene and the γb gene (Edwards, 1995). Similarly, for PsbMV seed transmission, HC-Pro (helper component-protease) was found to be a major determinant of seed transmission (Johansen et al., 1996). In pea early browning virus (PEBV), a 12-kDa polypeptide containing a cysteine-rich putative zinc finger structure with unknown function of RNA1 was identified as a viral determinant for seed transmission (Wang et al., 1997). Genetic variation in replicase and movement proteins of BSMV, CMV, and PsbMV was reported to be associated with the efficiency of seed transmission (Edwards, 1995; Wang et al., 1997). In TGMV, the viral factors required for mesophyll invasion from phloem were found to be BR1/BV1 expression, which requires the cis-acting BV1 and transacting AL2 factors through DNA *in situ* hybridization and genetic complementation studies (Sunter and Bisaro, 1992). Thus, it is necessary to observe for the involvement of BV1 and other genetic determinants in seed transmission of begomoviruses.

Resistance to seed transmission may happen from physical, physiological, and biochemical barriers for virus entry and replication in reproductive tissues. These barriers represent specific host-resistance mechanisms, which have either evolved to exclude viruses or merely as adaptation mechanisms. There is little experimental evidence available regarding the inheritance of resistance for seed transmission in plant–RNA virus system but not in begomoviruses. In barley cv. Modjo, in F_2_ population of a cross between susceptible and resistant barley lines to seed transmission of BSMV, resistance to seed transmission was found to be segregated as a single recessive gene (Carroll et al., 1979). Wang and Maule (1994) observed that resistance to seed transmission of PSbMV in *Pisum sativum* was inherited as a quantitative trait controlled by the action of multiple maternal genes. Similarly, resistance to AMV seed transmission was controlled by multiple genes in a quantitative manner in an F_2_ population obtained from two *Medicago murex* (L.) accessions (Pathipanawat et al., 1997).

The seed transmission of begomoviruses may occur either through direct or indirect pathway. The begomoviruses are the phloem-limited viruses and may exploit pore plasmodesmatal units/PDLP family to reach the reproductive structures via phloem vasculature. Then, the begomovirus may accumulate at a low level to escape the host surveillance mechanisms and suppress the host defense mechanism with the specific VSR proteins in reproductive tissues. In indirect invasion, begomoviruses may reach the ovary and MMC and presumably present in fully developed embryosac through maternal vascular connections by altering SEL of plasmodesmata. In direct invasion, begomoviruses may reach the embryo before the commencement of torpedo stage through suspensor. However, the virus has to encounter many barriers to reach the embryo post-fertilization. Here, the virus particles may follow the nutrient pathway to reach the filial tissues (embryo and endosperm), i.e., exit from sieve elements through plasmodesmata to the ovule where there may be some symplastic extension of the phloem present. Several studies showed that there was symplastic connectivity within and between the individual structures of mature seed, i.e., outer and inner integuments, endosperm, suspensor, and embryo at different developmental stages (Stadler et al., 2005; Werner et al., 2011). Thus, the virus particles may reach the seed apoplasm through seed coat vascular compartments embedded in the ground tissue where the plasmodesmata have large SELs (Patrik and Offler, 2001). Thereafter, the virus particles may reach the suspensor/embryo through extensive symplastic paths via interconnecting plasmodesmata. The presence of pore-like structures in the suspensor may facilitate the begomovirus movement to the embryo. However, it is necessary to analyze the process of begomovirus invasion into the floral organs to determine the presence of defense mechanisms restricting virus invasion, transcription factors supporting virus replication, and the motifs in the virus molecule regulating replication, accumulation, and trafficking in the flower primordia.

## Potential factors influencing seed transmission

The factors affecting virus seed transmission rates are complex and are the result of various interactions between the host, virus, vector, and environment (Maule and Wang, 1996). These factors are elaborated below:

- Host plant: different cultivars of the same host species can vary in their seed transmission rates for a particular virus. For example, TYLCV-IL was found to be seed transmitted in tomato cultivars Seogwang and Bacchus but not in Melillero, Rondeno, La Carlota, Zahara de la Sierra, Marmande, Cherry Canada, and Moneymaker (Kil et al., 2016; Perez-Padilla et al., 2020). This fact was already known long ago in RNA viruses, namely, PSbMV and BSMV. When PSbMV was tested in 38 pea cultivars, five of them exhibited no seed transmission (Stevenson and Hagedorn, 1973). For BSMV, the seed transmission rates reported to be varied from 0% to 75% in the different cultivars (Carroll and Chapman, 1970). The difference of seed transmission rates between genotypes might be due to the difference in timing of viral entry and abundance in the embryonic tissues. So far, limited information is available regarding begomovirus seed transmission rates in various cultivars of different crops. The comparison of seed transmission among different cultivars/genotypes will be helpful in understanding the important fact that seed transmission in begomovirus is a general or specific biological feature that is limited to specific genotypes of a plant species
- Virus isolate: different virus isolates vary with seed transmission rates even in a single host cultivar. These differences may reflect the virus isolate ability to replicate and move for successful invasion to gamete or the embryo (Carroll, 1981). The studies are lacking on seed transmission rate of various begomovirus isolates. However, in other viruses like BSMV, seed transmission of ND18 strain was found to be as high as 64% in ‘Dickson’ barley, whereas strain CV17 was < 1% in the same cultivar (Timian, 1974). BSMV seed transmission ranges from 0% to 100% for all BSMV strains (McKinney and Greeley, 1965). In PSbMV, isolate DPD 1(P-1) was highly seed transmitted, whereas NY isolate (P-4) was rarely seed transmitted (Johansen et al., 1996). It is necessary to identify the genome region responsible for seed transmission of begomovirus in the line, as it is known for whitefly transmission of begomovirus, which is located between the amino acid domains GCEGPCKVQS and LYMACTHASN or more specifically between amino acids 129 and 152 of the coat protein gene (Caciagli et al., 2009; Wei et al., 2014; Guo et al., 2018).
- Environmental factors: environmental factors can influence seed transmission by affecting the plant physiological state during seed development and maturation, changing the balance between virus multiplication, spread, and stability (Hanada and Harrison, 1977; Tu, 1992; Johansen et al., 1994). The conditions prevailed during embryo maturation, which ultimately affect virus stability and determine the final seed transmission rate (Maule and Wang, 1996). In CMV, seed transmission rate was decreased in lupin with the drought conditions with no significant effect on grain yield (Jones and Proudlove, 1991). Several reports have stated that lower temperatures favor vertical/seed transmission, while higher temperatures increase virus titers (Frosheiser, 1974; Tu, 1992). The studies using SMV elucidated that virus symptoms on the mother plants were most severe when grown at 25°C, seed transmission was optimal when grown at 20°C (average 48%), and seed transmission decreased at 15°C (average 7%) and 25°C (average 9.7%) (Tu, 1992). The work with PSbMV showed that lowered rainfall reduced the virus incidence in the field, thereby seed transmission (Coutts et al., 2009). In turnip mosaic virus, it was observed that a higher light intensity increased the efficiency of seed transmission in *Arabidopsis* through modification of environment-related plant resistance and tolerance (Montes and Pagan, 2019). Thus, the risk associated with a given level of seed infection mainly depends on the conditions before and after planting under field conditions. Furthermore, differences in growth conditions such as greenhouse versus growth chamber cultivation drastically affect the efficiency of seed transmission process as observed in TYLCV (Rosas-Diaz et al., 2017). Hence, the emergence of begomoviruses seed transmission might be due to the potential of climate change conditions in modifying the outcome of plant–virus interactions and contributing to seed transmission.
- Seed development and longevity: it is speculated that seed transmitted viruses could favor a long period of seed fertility to ensure them to be transmitted to the plant progeny. This statement is consistent with the life-history theory that states that interaction with low virulent parasites will result in a delay in host reproduction, which allows for compensation of parasite damage (Gandon et al., 2002). The presence of virus in the seed reduces its viability and thereby leading to lower long-term seed survival from infected plants. Hence, there is a negative relationship between efficiency of seed transmission and long-term seed survival as observed in the case of CMV infection on *Arabidopsis* (Cobos et al., 2019). It is believed that a virus that has infected the embryo will remain viable for as long as the seed is viable as observed in the case of BCMV where it survived and remained infectious for 30 years in seed (Pierce and Hungerford, 1929; Bennet, 1969). Such studies are yet to be conducted with begomoviruses to estimate their survival period in seed.
- Virus virulence and seed transmission: the central hypothesis states that vertically transmitted pathogens evolve reduced virulence and horizontally transmitted pathogens evolve moderate or high levels of virulence (Clayton and Tompkins, 1994; Messenger et al., 1999). There are controversial reports regarding the virus virulence and seed transmission. The fitness of vertically transmitted viruses is highly dependent on host reproductive potential, as hosts need to reproduce for the virus to infect new individuals. In most of the plant–virus interaction studies, virus virulence was found to be negatively correlated with vertical transmission (Kover and Clay, 1998; Stewart et al., 2005). In BSMV, it was shown that increased horizontal transmission was often due to increased virulence, and increased vertical transmission was due to reduced virulence (Stewart et al., 2005). The seed transmission experiments with CMV in *Arabodopsis* for five generations indicated that vertical passaging led to adaptation of the virus to greater vertical transmission, which was associated with reduction in virus accumulation, multiplication, and virulence (Pagan et al., 2014). In begomovirus, this biology is yet to be established.
- Timing of infection: to ensure seed transmission, it is critical that the virus should reach and invade plant reproductive organs before gametogenesis and/or while the embryo is still accessible from the mother plant without affecting gamete/embryo viability. For begomovirus, the data for time of infection and time of accessibility in the reproductive organs are lacking. However, such information is available in the case of barley flower tissues, where the seed- and pollen-transmitted BSMV isolate MI-1 invaded the floral primary meristem early and subsequently to the PMC, sperm, and the MMC including the egg (Carroll and Mayhew, 1976a, 1976b). Studies with PEBV-infected pea showed that the virus was present in the synergids of the egg cells, in the polar nuclei from unfertilized ovules, and in the mature pollen from unopened flower anthers (Wang and Maule, 1997). Prunus necrotic ringspot virus (PNRSV) was shown to invade early the pollen grains, generative cell of the bicellular pollen grain, and megaspore in apricot trees (Amari et al., 2007). Thus, early positioning of PNRSV at the pollen tube emergence apertures followed by growing tips could increase the transmission opportunities of this virus during fertilization (Amari et al., 2009).
- Viral synergism: synergism can influence virus seed transmission rates, although the direction of effect appears to vary. In begomoviruses, the effect of viral synergism on seed transmission has not yet reported. A synergistic effect that increases the seed transmission rate is known for viruses in other families, *viz*., SBMV and turnip yellow mosaic virus (Kuhn and Dawson, 1973; De Assis Filho and Sherwood, 2000).

## Epidemiological significance of *begomovirus* seed transmission

**Figure 2:**
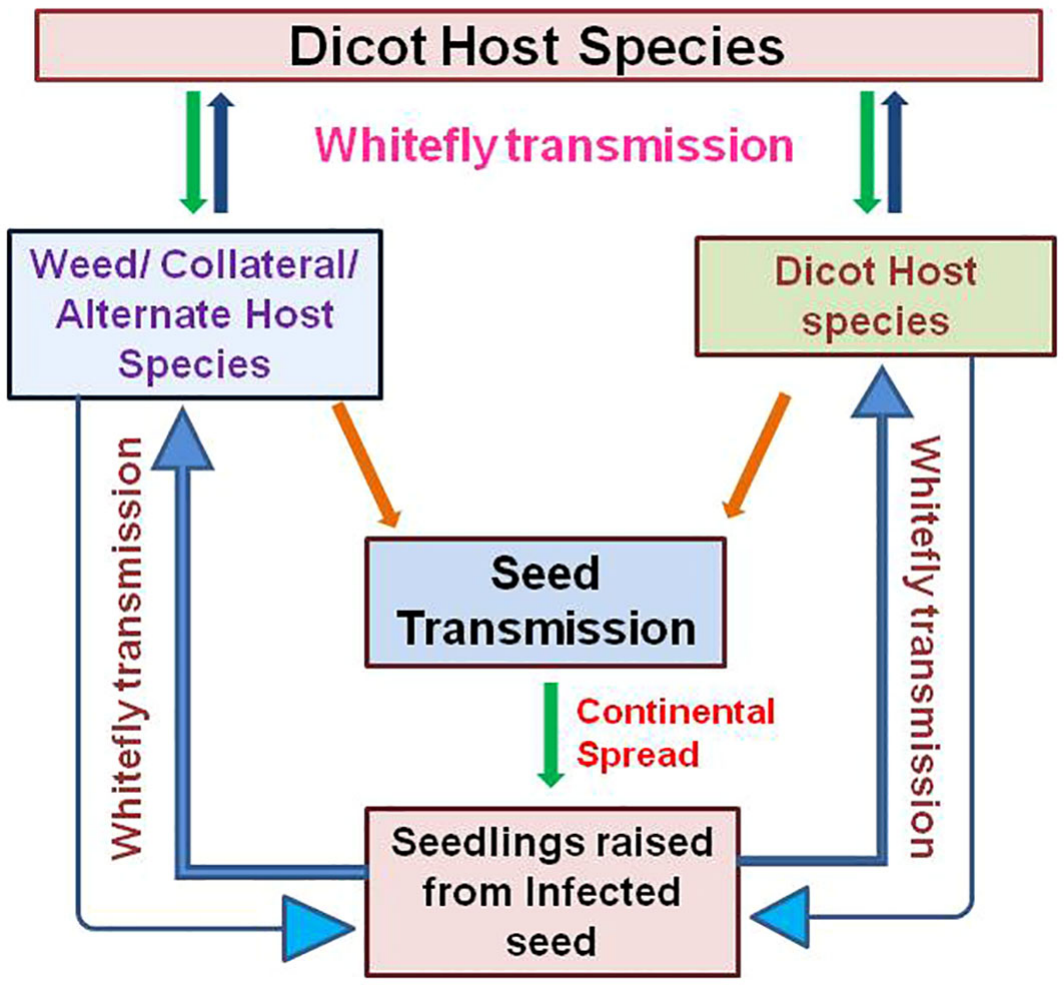
Schematic representation of epidemiological significance of begomovirus seed transmission in the circulation and outbreak of the disease along with extended host range.

Seed transmission is an important factor in the epidemiology of plant viruses, as it helps virus survival during unfavorable climatic conditions and spread beyond the limits of time and place where vector would not reach and may have considerable impact on crop yield (Albrechtsen, 2006; Ali and Kobayashi, 2010; Fabre et al., 2014; Pagan, 2022). In the case of begomoviruses, seed transmission has emerged as a major concern because it is mostly in the form of seeds that the germplasm collections are conserved and exchanged internationally (Mink, 1993; Sastry, 2013). Begomovirus in seed will have a serious implication in the circulation and outbreak (**Figure 2**).

- Circulation of virus: seed transmission can be an important source of early and randomized primary infection foci within a crop field from which the virus spreads subsequently to other plants through vector transmission. These plants will subsequently act as secondary source of infection depending on their susceptibility (Maule and Wang, 1996; Simmons and Munkvold, 2014). Hence, sowing of the begomovirus-infected viable seed can introduce potent primary infection within the growing stand from where the whitefly can acquire and spread the disease rapidly to healthy plants, as shown in the case of TYLCV and ToLCNDV (Kil et al., 2016; Gomathi Devi et al., 2023). As most of the cultivated, wild alternative, and weed species are the hosts for begomoviruses, seed transmission can help the virus to persist in these plant populations, which can further act as reservoirs (Regassa et al., 2021). Recent studies reported the expansion of begomovirus host range to monocotyledons, the movement of *B. tabaci* vector to temperate regions, and the transmission of begomoviruses by whiteflies other than *B. tabaci* complex (Idriss et al., 1997; Kriticos et al., 2020; Kil et al., 2021; Fiallo Olive and Navas-Castillo, 2023). In such cases, seed-transmitted novel begomovirus variants and species may spread across the globe through seed trade and insect vectors and responsible for epidemics in host and non-host species under changing global climate scenario. Furthermore, seed infection provides the begomovirus with the means to persist for long periods of time when hosts and vectors are not available, as shown in other seed-transmitted viruses (Bos, 1977; Simmons and Munkvold, 2014). This facilitates virus emergence and re-emergence in plant populations (Hamelin et al., 2016; Pagan, 2019).
- Outbreak of disease: virus transmission process is one of the most important factors in understanding a plant virus outbreak and its epidemiology. Seed transmission in begomoviruses is being perceived to have an igniting potential in epidemic outbreak. This is due to high vectoring efficiency, and abundance of whitefly can compound into successive flare up of begomovirus disease from the primary foci of infection brought in to the field through seed transmission. Seed transmission of begomoviruses even at very low rates is critical for the spread, overwintering, and long-distance dissemination of viruses. However, the seed transmission rate is not necessarily a sole indicator for outbreak of the disease that leads to epidemics. Low seed transmission rate in conjunction with secondary spread by the vector can produce disease epidemics, as observed for LMV with 0.001% seed transmission along with subsequent spread by aphid vector (Ryder, 1973; Dinant and Lot, 1992). In the case of chilli leaf curl virus, a significant begomovirus in the Indian subcontinent, it was shown that a small increase in immigration rate of viruliferous whitefly increased the population of both infected plants and viruliferous vector population that, over a short period of time, compounded in rapid spread of the virus within the field (Roy et al., 2021 & 2023). When seed transmission provides primary foci of begomovirus infection in the field, the disease outbreak is imminent with the abundant prevalence of whitefly as observed for ToLCNDV. In bitter gourd, it was observed that the initial infection was 43% due to ToLCNDV seed transmission, which increased up to 72% after the release of whiteflies (Gomathi Devi et al., 2023). Thus, when seed transmission provides initial primary foci, the disease outbreak can occur when conditions influencing the virus, host, and its vector synchronize along with efficient build-up of virus inoculum, which ultimately lead to development of an epidemics. Thus, begomoviruses can initiate damaging epidemics even at lower seed transmission rates.

To understand the epidemiological consequence of begomovirus seed transmission, extensive experimental work should be carried out with close observation on the virus, crop, and weed species along with vector population present in a particular area that helps to predict future outcomes (Duffus, 1971; Regassa et al., 2021). The actual outcome when the infected seed with a given level of infection is sown depends upon the impact of climate, soil, and biological factors on whitefly population and virus spread. Hence, there is an emergency to determine the acceptable ‘threshold’ levels of infection in seed stocks with different levels of begomovirus seed infection by considering the factors like germination and survival of seed-infected plants, virus disease progress during cropping season, magnitude of yield loss, and amount of infection in harvested seed (Jones, 2000). The development of simulation/forecasting models by involving seed transmission, host species, whitefly migration, immigration, and emigration along with climatic factors can extend the understanding of begomovirus disease epidemics due to seed transmission (Sastry, 2013).

## Management strategies to reduce the impact of *begomovirus* seed transmission

The impact of begomovirus seed transmission can be minimized through routine seed health testing, production of disease free seed, cultural practices, development of genetic resistance to seed transmission, and to a lesser extent with heat and chemical treatment of infected seeds (Aveling, 2014; Pagan, 2022). The advanced seed health testing methods are of little value unless the actual threshold levels of virus infection has been determined (Jones, 2000; Coutts et al., 2009). In most of the plant viruses, the acceptable threshold level of seed infection remains unclear. For example, the acceptable threshold limit of LMV-infected lettuce seed was 0.1% where it was shown that even 0.001% could start an epidemic (Ryder, 1973). Seed health tests can become reliable for the prediction of disease development and dissemination of pathogen only if we will take into account the inoculum density and environmental pressure.

Regeneration of virus-free plants from virus-infected individuals through meristem culture has been increasing and applied to many commercial crops (Panattoni et al., 2013). Virus exclusion from the meristematic cells might be due to the cellular preclusion of the virus and partially through existence of sequence-specific RNAi-dependent mechanisms (Bradamante et al., 2021). Thermotherapy is a method based on heat treatment of infected seeds at temperatures between 35°C to 54°C for a specific period of time based on physiological tolerance limit (Spiegel et al., 1993; Aveling, 2014). Thermotherapy may reduce virus load but rarely result in complete eradication along with compromised seed viability (Paylon et al., 2014). Chemical disinfectants like sodium hypochlorite, trisodium phosphate, and hydrochloric acid may help in removing the viruses present on the seed coat (Paylon et al., 2014). Even though chemotherapy is well known for virus elimination by using ribavirin and virazole, the studies were limited (Huffman et al., 1973).

Cultural practices like avoidance of continuous cropping especially in legumes can break the disease cycle and thereby minimize the spread of virus diseases, which have limited host range like MYMV and MYMIV (Sastry, 2013). The elimination of weed, volunteer and wild hosts that act as a direct source of viruses, reduces the source of infection and virus spread in seeds and thereby reduces the chance of epidemics development within the crops (Regassa et al., 2021). Rouging of diseased plants from main crop and crop rotation practices to eliminate the virus-infected volunteer plants can help in minimizing the spread of virus, and thereby, disease can be managed. Quarantine is one of the most effective measures for preventing the movement of seed transmitted viruses (Rubio et al., 2020).

Employment of specific genetic resistance to seed transmission will be the best strategy to manage seed-transmitted begomoviruses even though it has limited durability (Garcia-Arenal and McDonald, 2003; Pagan, 2019). Genetic resistance could reduce the impact of seed transmission in virus epidemics through production of virus-free certified seeds, thereby reducing the sources of primary inoculum. Cultivar-specific resistance to seed transmission has already been reported for AMV in alfalfa, BSMV in barley, PsbMV in pea, and SMV in soybean (Johansen et al., 1994). However, this strategy requires extensive knowledge on the molecular mechanism underlying seed transmission and resistance to infection of the seed tissue on the mother plant. A new approach CRISPR-Cas (clustered regularly interspaced short palindromic repeats) can be exploited for the development of resistance in the cultivars to seed transmission (Barrangou et al., 2007), as this method was already successfully reported for virus resistance against begomoviruses (Ali et al., 2016; Tashkandi et al., 2018; Roy et al., 2019).

## Conclusions and future prospects

The centrality of understanding the natural process of begomoviruses transmission exclusively through whitefly, *Bemisia tabaci*, have changed with the current findings of seed transmission in some begomoviruses. As a result, in recent years, a strong interest on seed transmission, a new domain of begomovirus research, has emerged. Various studies on seed transmission of begomoviruses cleared that seed transmission is not a general property of begomoviruses. An interesting aspect of begomovirus seed transmission is the overlapping host range especially seed transmission in cucurbitaceous crops, as reported for ToLCNDV and TYLCV seed transmission in asymptomatic hosts sweet pepper and soybean. This sudden possibility of seed transmission of begomoviruses might be due to recombination events between begomoviruses and synergistic effect of multiple infections resulting in the emergence of seed transmitted forms of begomoviruses.

Seed transmission of begomoviruses is a major concern for seed production, seed industries, and international trade, which may warrant the adaptation of regulatory measures. Hence, it is crucial to confirm the earlier findings of begomovirus seed transmission through efficient diagnosis and whitefly transmission studies to understand the effect on disease epidemiology. While many questions are yet to be answered, the role of begomovirus proteins in ensuring seed transmission, mechanism of virus movement from vegetative to reproductive tissues of host plant, role of host defense mechanisms in suppressing the seed transmission, and latency of begomovirus during seed maturation environment are immediate important areas of understanding for the establishment of the seed-borne nature of begomoviruses. There are many examples of the presence of begomovirus in the seeds derived from infected plants; however, often plants developed from such seeds were asymptomatic. The basis of such biology is yet to be understood. There is an urgent need to study the ecology and biology of seed-borne begomoviruses in relation to climate change.

Once the begomovirus seed transmission will be established, then strict biosecurity and seed health regulations need to be enacted promptly and adopted rigorously. It is necessary to establish sound threshold level of seed-borne begomovirus infection that can be used to advise growers, regulators, breeders, and commercial seed industry. Establishment of an effective surveillance and monitoring system for seed-transmitted begomoviruses can help in preventing the spread from endemic to non-endemic areas. Practicing of community-based approach by seed companies along with implementing standard operational procedures for virus-free seed production can somewhat reduce the damage caused by seed-borne begomoviruses. Development of cost-effective harmonized diagnostic protocols for the detection of seed-borne begomoviruses in commercial seed lots can help in designing effective management strategies under changing climate scenario.

## Author contributions

NS: Writing – original draft, Writing – review & editing. BM: Conceptualization, Writing – review & editing.
